# Revealing the complexity of vampire bat rabies “spillover transmission”

**DOI:** 10.1186/s40249-023-01062-7

**Published:** 2023-02-13

**Authors:** Luis E. Escobar, Andres Velasco-Villa, Panayampalli S. Satheshkumar, Yoshinori Nakazawa, Paige Van de Vuurst

**Affiliations:** 1 Department of Fish and Wildlife Conservation, Virginia Tech, Blacksburg, VA USA; 2 Virginia Tech Graduate School, Translational Biology, Medicine, and Health Program, Blacksburg, VA USA; 3 Global Change Center, Virginia Tech, Blacksburg, VA USA; 4 Center for Emerging Zoonotic and Arthropod-Borne Pathogens, Virginia Tech, Blacksburg, VA USA; 5 Facultad de Ciencias Agropecuarias, Universidad de La Salle, Bogotá, Colombia; 6 Poxvirus and Rabies Branch, Centers for Disease Control and Prevention, 1600 Clifton Rd. NE, Atlanta, GA 30333 USA

**Keywords:** Bat-borne diseases, *Desmodus rotundus*, Rabies, Spillover, Transmission

## Abstract

**Background:**

The term virus ‘spillover’ embodies a highly complex phenomenon and is often used to refer to viral transmission from a primary reservoir host to a new, naïve yet susceptible and permissive host species. Spillover transmission can result in a virus becoming pathogenic, causing disease and death to the new host if successful infection and transmission takes place.

**Main text:**

The scientific literature across diverse disciplines has used the terms virus spillover, spillover transmission, cross-species transmission, and host shift almost indistinctly to imply the complex process of establishment of a virus from an original host (source/donor) to a naïve host (recipient), which have close or distant taxonomic or evolutionary ties. Spillover transmission may result in unsuccessful onward transmission, if the virus dies off before propagation. Alternatively, successful viral establishment in the new host can occur if subsequent secondary transmission among individuals of the same novel species and among other sympatric susceptible species occurred. As such, virus spillover transmission is a common yet highly complex phenomenon that encompasses multiple subtle stages that can be deconstructed to be studied separately to better understand the drivers of disease emergence. Rabies virus (RABV) is a well-documented viral pathogen which still inflicts heavy impact on humans, companion animals, wildlife, and livestock throughout Latin America due substantial spatial temporal and ecological—natural and expansional—overlap with several virus reservoir hosts. Thereby, the rabies disease system represents a robust avenue through which the drivers and uncertainties surrounding spillover transmission can be unravel at its different subtle stages to better understand how they may be affected by coarse, medium, and fine scale variables.

**Conclusions:**

The continued study of viral spillover transmission necessitates the elucidation of its complexities to better assess the cross-scale impacts of ecological forces linked to the propensity of spillover success. Improving capacities to reconstruct and predict spillover transmission would prevent public health impacts on those most at risk populations across the globe.

**Graphical Abstract:**

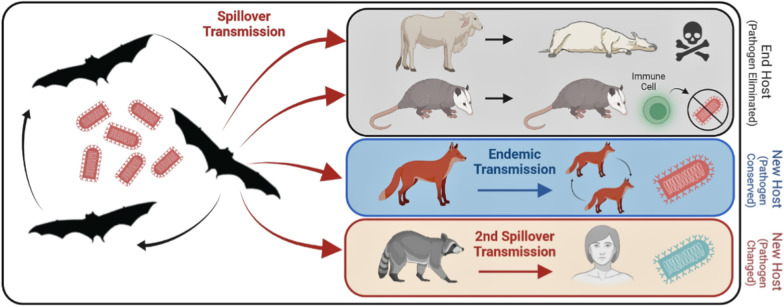

## Background

Viruses have interacted with their hosts for at least thousands of years [1]. Indeed, most viruses are expected to be host-specialists—infecting a single host species, instead of host-generalists—infecting multiple host species [2]. This long-term host-virus relationship allows the virus to induce negligible damage to its host in a process-termed co-evolution [3]. Thus, host-virus co-evolution facilitates coexistence by reducing reciprocal harm: viruses modulate their virulence against the host and hosts modulate their immune response against the virus [4]. A stable host-virus relationship, however, can be disrupted by external forces, causing the manifestation of disease (i.e., detrimental effect of the virus over the host). Cross-species virus transmission, from the original host to a new host, occurs in fish, plants, and wildlife, which has also been referred as spillover transmission. Using an ecological framework, this comment article reveals the inherent complexities of *spillover transmission*, strictly defined as the processes that allow cross-species transmission of viruses causing disease (i.e., pathogens) in humans (i.e., zoonotic pathogens; Box 1). Nevertheless, the rationale could be useful for non-zoonotic and non-directly transmitted viruses and other infectious agents.

More than 75% of emerging infectious diseases in humans have originated from spillover transmission events between hosts that do not share obvious evolutionary histories [5]. Thus, it has been proposed that evolutionary biology alone has failed to anticipate emerging infectious diseases [6]. This failure could be due in part to the confirmation bias of spillover transmission studies which have been historically focused on (1) viruses that had successfully established in a naïve host causing the emergence of novel transmission cycles, (2) viruses that are virulent to a naïve yet susceptible host causing conspicuous disease, and (3) zoonotic viruses [7–10]. Nevertheless, during spillover transmission events wildlife viruses are not guaranteed to establish in a naïve host, and may or may not affect humans in a negative way [10]. Thus, identifying or revealing factors at different scales of complexity associated with spillover transmission is key for the better understanding and forecasting of disease emergence.

### Defining spillover transmission

**Fig. 1:**
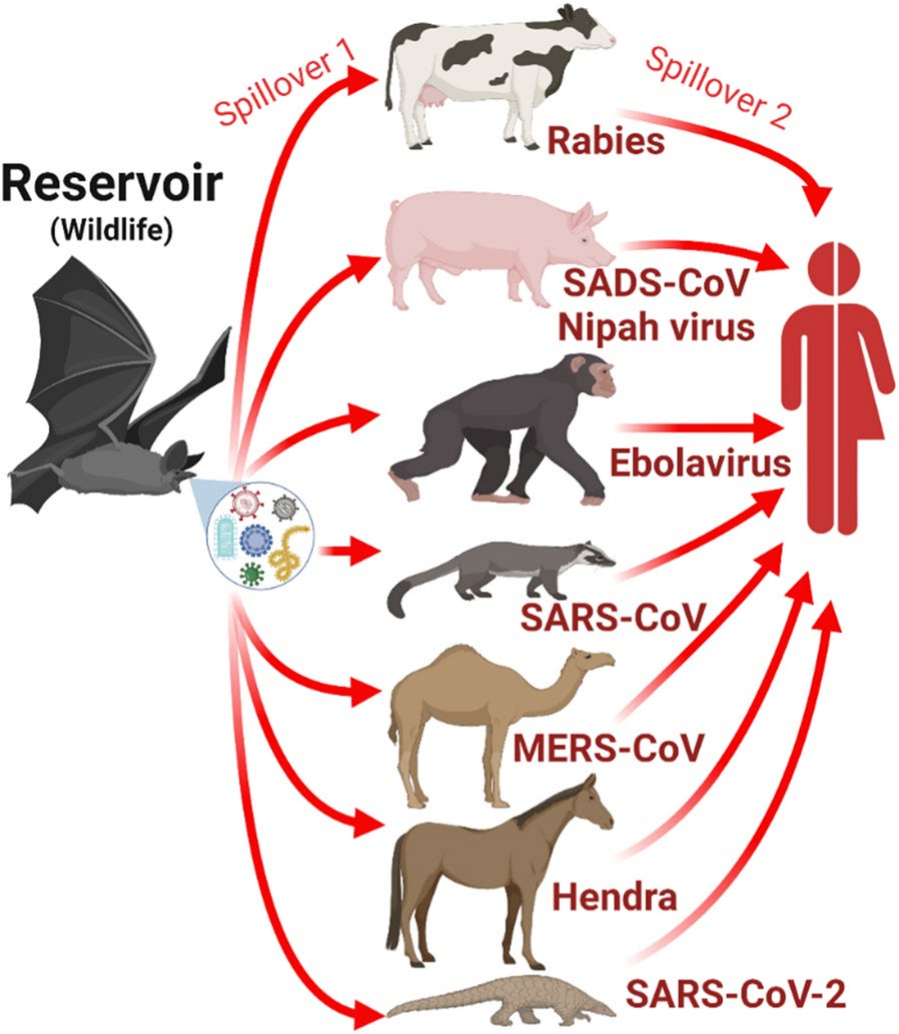
Recent spillover transmission events. A native reservoir serves as the source of a virus to other host species, in which it could be virulent. This accidental cross-species transmission (i.e., spillover transmission) (red arrows), occurs between host species without evolutionary relatedness. Spillover transmission between two genetically unrelated species has been documented in recent emerging infectious where a pathogen from an original wildlife host (left) is transmitted (*primary spillover*; red arrows on the left) to another wildlife or domestic species (middle), and from them to a human (*secondary spillover*; right). SADS-CoV: swine acute diarrhea syndrome coronavirus. SARS-CoV: severe acute respiratory syndrome coronavirus. MERS-CoV: Middle-East respiratory syndrome. SARS-CoV-2: severe acute respiratory syndrome coronavirus 2

The transmission of a virus from one species to another is termed “*spillover transmission*” [10, 11]. As such, “virus spillover” is a correct term commonly used in epidemiology of zoonoses to refer to cross-species or interspecies transmission events. “Disease spillover”, in contrast, is an incorrect term commonly used in the gray literature, to refer to the same phenomenon. Disease spillover is an erroneous use of the spillover term because diseases per se cannot be transmitted, only their causative agents (e.g., viruses). Many emerging infectious diseases have originated via spillover transmission of viruses from an original or primary wildlife hosts (i.e., reservoir host) to new, naïve domestic animal hosts followed by successful onward intraspecific transmission (Fig. 1). In some instances, wide host-range viruses transmitted from wildlife to domestic animals can reach humans through a secondary spillover transmission event (i.e., spillover transmission from new hosts, instead of the original host). For example, outbreaks from spillover transmission in the last two decades include: rabies virus (RABV; *Lyssavirus*) from vampire bats to cattle in Latin America [12, 13], swine acute diarrhea syndrome coronavirus (SADS-CoV; *Alphacoronavirus*) from *Rhinolophus* spp. bats to pigs in China [14], Nipah virus (*Henipavirus*) from flying-fox bats to pigs in Bangladesh [15, 16], Marburg virus from the African fruit bat (*Rousettus aegyptiacus*) to primates in Africa, severe acute respiratory syndrome coronavirus (SARS-CoV or SARS-CoV-1; *Betacoronavirus*) from bats to palm civets [17], Middle-East respiratory syndrome (MERS-CoV; *Betacoronavirus*) from bats to camels in the Middle East [18–20], Hendra virus (HeV; *Henipavirus*) from flying-fox bats to horses in Australia [21], and severe acute respiratory syndrome coronavirus 2 (SARS-CoV-2; *Betacoronavirus*) from bats to a yet unknown secondary host in Southeast Asia [22–25] (Fig. 1).

**Fig. 2:**
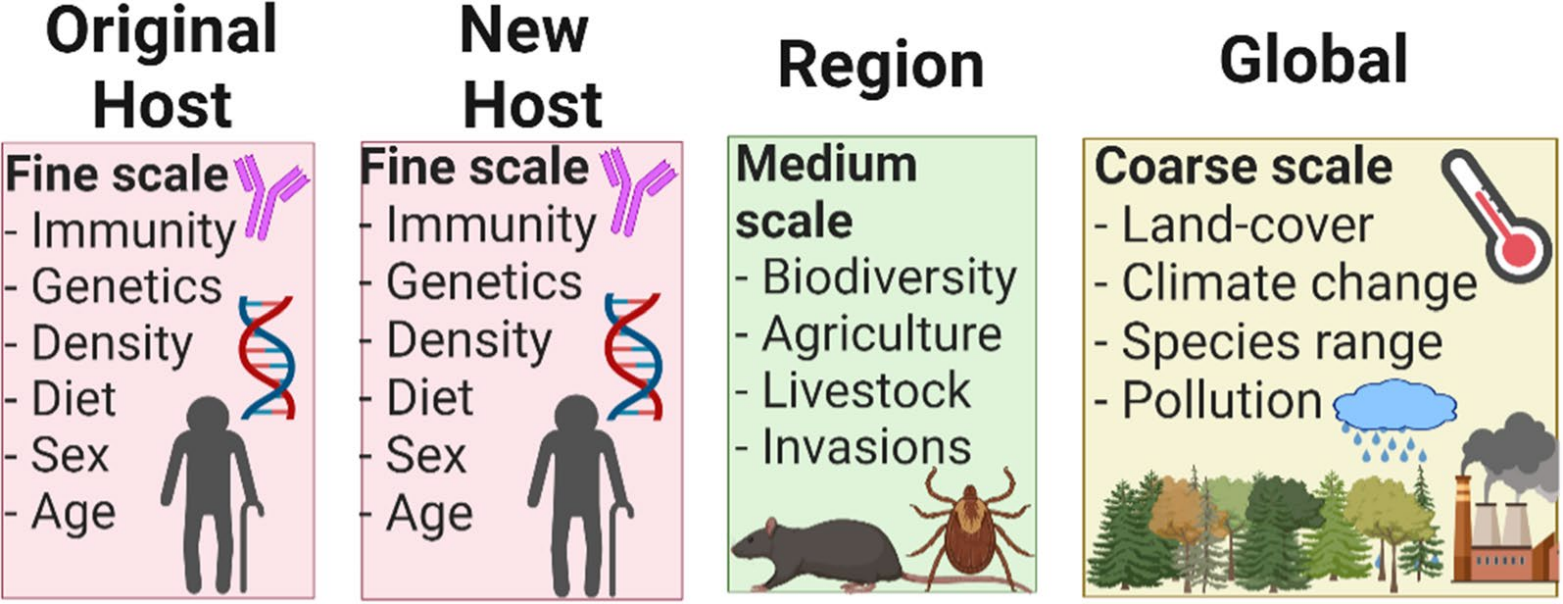
Factors potentially related to spillover transmission across scales. Different biological process act at different spatial scales and may have variables impacts across those scales. Which scale is appropriate to study specific patterns and parameters is still an unresolved question in disease ecology. Studies at fine scale provide high-detail and specificity, but cover small areas while coarse-scale studies cover large geographic areas at the cost of detail

Strikingly, there are no known empirical indicators for predicting the likelihood of spillover events. Nevertheless, spillover transmission events are better characterized for some viruses, such as bat-borne rabies in Latin America, which offers opportunities to better understand spillover transmission and successful onward transmission to secondary susceptible hosts. A series of factors could contribute to the likelihood of spillover transmission and successful virus establishment across scales, from micro to macro. At the fine scale, susceptibility, immune status, genetics, population density, availability of resources (e.g., diet), sex, and age of the host reservoir could be linked to higher levels of viral shedding or higher contact rates to increase spillover transmission and successful virus establishment in a new species (Fig. 2). At an intermediate scale, biodiversity composition, expansion of agriculture, and intensification of livestock production could play a role in the likelihood of spillover transmission by facilitating interactions between species. Finally, at coarse scales, global climate and landscape change or expansion of species ranges could also influence the risk of spillover transmission.

Furthermore, factors facilitating spillover transmission may act across scales and be interconnected. For example, paleontological evidence suggests that extinctions of large mammals due to climate change increased the endemicity of pathogens in the remaining wildlife community, which may conduce to increased spillover transmissions from wildlife to humans [26]. Furthermore, climate and landscape variation modulate mammal species composition, which shapes virus speciation and endemism altering viral spillover rates between primary hosts and sympatric naïve hosts [27–29]. As such, biodiversity losses due to global change could influence the propensity of viral spillover transmission [30], evidenced by previous studies on the influence of potential changes in wildlife species assemblages due to environmental change [31–34].

### Spillover transmission versus disease emergence

**Fig. 3:**
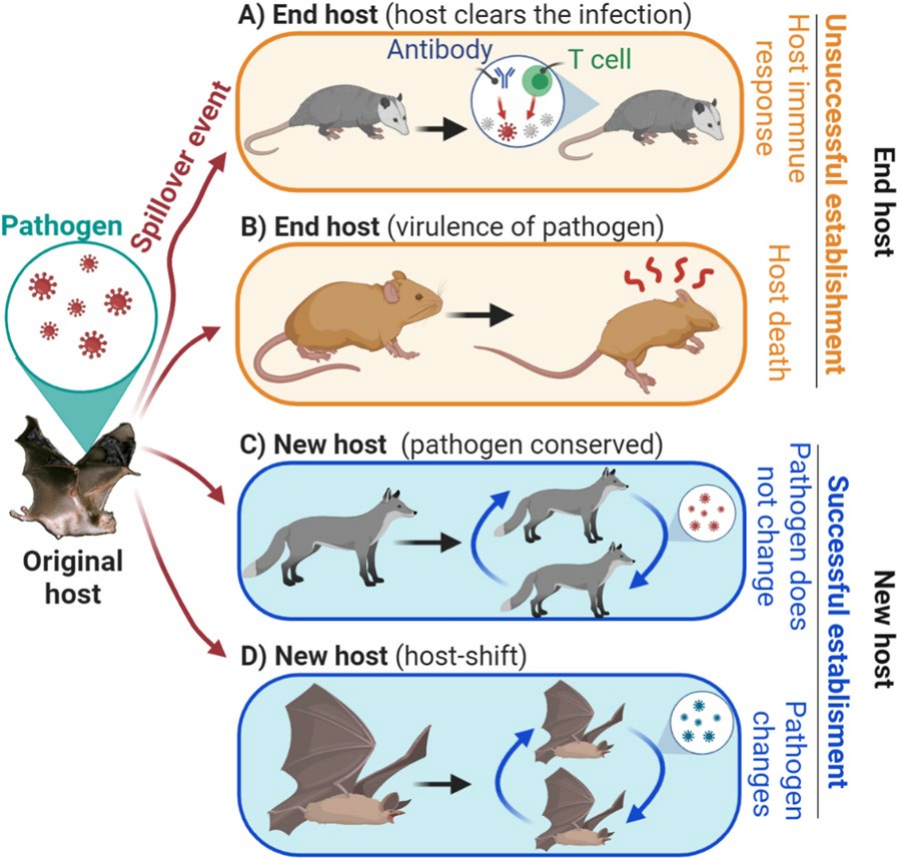
Diagram of spillover transmission events with successful and unsuccessful virus establishment in the new host. A geographical range shift of a host species may cause contact with a novel host species. This disturbance in host species assemblages may cause transmission of viruses from the primary host to a new, naïve host, resulting in the expression of disease or death of the new host. Alternatively, the new host infected with the virus could show complete innocuity due to the incompetence of the host to interact with the novel virus. **a** Pathogens generally fail to successfully establish in a naïve host (orange) due to the virus removal (e.g., the immune system clears the infection or there is no cell tropism compatibility to lack of affinity with cell receptors). **b** Pathogens could also fail to establish in a new host due to the host’s death (e.g., pathogen kills the naïve host). Failure of the pathogen to establish in a new host, also known as ‘end hosts’ (orange), will not generate secondary transmission. **c** In rare spillover transmission events, pathogens successfully establish in a naïve host in the absence of pathogen’s adaptation (Pathogen does not change; i.e., pathogen’s complete genome highly conserved, without adaptative mutations. Under this scenario pathogens mainly present neutral evolution). **e** Alternatively, the pathogen could develop adaptation (i.e., mutations) to modulate its virulence and escape or overcome the host’s immunity or increase cell receptor affinity (Pathogen change, i.e., host-shift). Successful pathogen establishment in a naïve host, also known as ‘new host’ is the prelude for disease emergence

Pathogen virulence and pathogen-induced extinction risks are generally considered to be low in reservoir hosts [35]. Nevertheless, in the new host spillover transmission can have two different outcomes: successful and unsuccessful establishment (Fig. 3). In unsuccessful virus establishment, the virus fails to establish in the new host due to factors such as the new host having no cell receptor affinity [36], low intracellular compatibility (e.g., no compatible codon usage) [37], a robust immune response that clears the infection, or having behaviors which reduce exposure to the virus (e.g., solitary vs. social species) [38]. Similarly, the virus could fail to establish due to high virulence in the new host species, which results in the death of the infected host before sustained propagation or onward transmission among conspecifics can occur. New hosts which do not generate secondary infections or onward transmission among conspecifics during spillover transmission are termed “end hosts.” Alternatively, viruses may successfully establish and generate brief or sustained onward transmission, resulting in a “new” reservoir host. For example, new hosts could have an immune response which is insufficient to clear the infection, allowing the virus to multiply and be transmitted onward to new individuals of the same species before the death of the new individual host. Establishment of the virus can be achieved without the need for viral evolution or adaptation—“virulence conservatism”—or reflected as evolutionary change in the virus—“host shift”. Secondary onward transmission resulting from successful viral establishment, either with or without viral evolution, is the prelude to disease emergence [10, 39–46]. An important knowledge gap that has not yet been fully explored with regard to disease emergence includes the role of habitat conditions or biodiversity gradients that may limit or facilitate secondary onward transmission (Box 1).

Box 1 Defining spillover In this opinion article, spillover transmission does not necessarily implies onward transmission after the new host is infected. Instead, spillover transmission refers to an specific moment in space and time when a given viral agent established in its primary reservoir host, circumstantially meets a new host. Thus, spillover transmission can be strictly referred as the moment of encounter, which implies a virus, somehow, enters a new host. Nevertheless, entering a new host does not necessary implies a virus will be successful to establish an infection and therefore be able to get transmitted intra-specifically (i.e., among individuals of the same species) or inter-specifically (i.e., among individuals of different species). Both scenarios denote onward transmission, though with some subtle differences.

Once spillover transmission occurs (the encounter), a onward transmission will depend on a successful infection (i.e., establishment of the virus in the new host). A successful infection will only happen if the virus finds an adequate permissive target cell and the virus possesses effective tools or mechanisms to evade the innate and adaptative host’s immune response of the host. Spillover could be considered as an stochastic event that may be governed by the probability a new host could get in touch with a virus from another species, regardless the virus’ ability to cause a successful infection with a consequent onward transmission

### Spatial scale

**Fig. 4:**
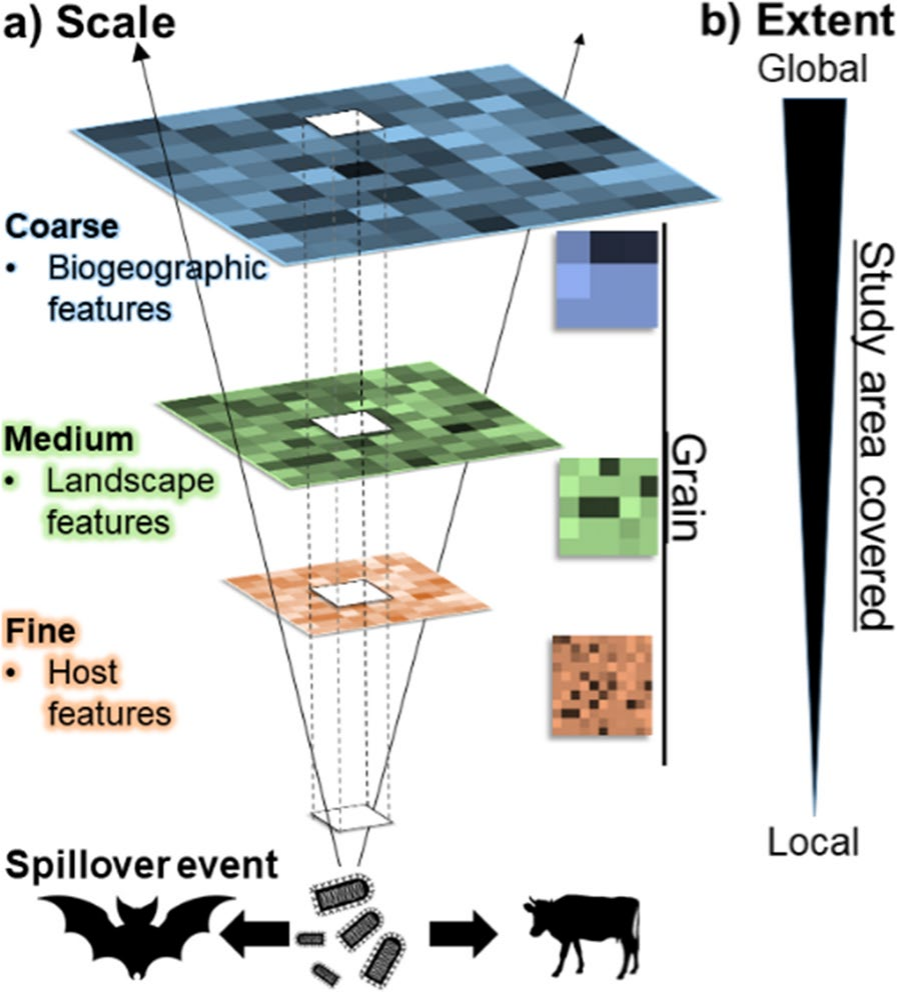
Multiscale framework of spillover research. **a** Pathogen spillover studies across fine, medium, and coarse scales allow bottom-up and top-down assessments. **b** Different scales allow to cover different study area extents, from individuals, to ecosystems. Modified from [74]

Different biological process act at different spatial scales [47]. For example, while behavior may be relevant to explain transmission patterns at the local level, climate could be more important to explain transmission at the continental level [48]. The appropriate scale to study specific patterns and parameters is still an unresolved question in disease ecology [49, 50]. Studies at fine scale provide high-detail and specificity but cover small areas, while coarse-scale studies cover large geographic areas at the cost of detail (Fig. 4). Spillover and onward transmission from an original host to a naïve host has been studied at the molecular, individual, and population level. Nevertheless, there is a need for spillover-transmission research at coarser scales to untangle the biogeographic elements of cross-species transmission [51]. Different spatial scales allow for the use of data with different information and at different levels of detail (i.e., grain; Fig. 4). At fine spatial scales, spillover studies can analyze host-level data (e.g., sex, age, genetic variation, body size, etc.) to assess the role of reservoir features on spillover risk. At medium spatial scales, researchers should assess landscape-level data (e.g., land cover change, intensity of urbanization, livestock density) to understand these features’ impact on spillover propensity. At coarse spatial scales, future investigations should explore how biogeographic factors (e.g., temperature, precipitation, evapotranspiration) explain why virus spillover transmission occurs in some areas but not others and when onward transmission is more likely.

### Bats and spillover

Bats are known to be major reservoirs hosts for many viruses, and consequently the primary source (donor or source host) of microorganisms that can successfully establish in naïve species (recipient host) [52, 53]. The high number of bat species (~ 1200) inherently generates a proportionally high amount and diversity of viruses (virosphere) with the potential to infect humans and other mammals [54]. That is, more bat-borne viruses are expected to be discovered as research effort increases [55]. As such, global hotspots of bat diversity are expected to harbor a larger and more diverse plethora of microorganisms with pandemic potential.

Viral establishment in the new host is dependent upon the frequency of spillover transmission events and demography of the new host [35]. Intermediate levels of virulence are also highly suitable for successful establishment in a naïve host species [35]. Pathogen virulence generally dependents upon the host affected [36] (Fig. 3). That is, a virus could be highly virulent for some host species or individuals, but not virulent for others. Molecular-level mechanisms of infection are well understood for many emerging diseases originated from spillover [3]; thus, one next frontier on spillover transmission research is a focus on the drivers of the spillover events themselves to understand why spillover occurs, which is neglected in many spillover transmission studies.

### Rabies as a model of spillover transmission

Rabies is caused by all members of the *Lyssavirus* genus, and ranks among the best-understood and best-surveyed disease systems [56, 57]. RABV is the most widespread agent within the genus and its detection and identification are achieved with high certainty by continent-wide comprehensive surveillance systems [58]. RABV is distributed across the globe, with hotspots of viral diversity along the Neotropics [59, 60]. Rabies virus transmission requires direct contact, through bites and scratches [61], and distinct viral lineages have become established in different species and populations within the Carnivora and Chiroptera orders [55]. Furthermore, the study of rabies was foundational to the development of the idea of vaccination (i.e., by Louis Pasteur); modern molecular epidemiology; disease eradication under One Health approaches; development of oral vaccination strategies; and for understanding disease persistence, natural immunity, abortive infections, spillover transmission, disease ecology, and evolution [57, 61]. Rabies is also almost 100% fatal for infected individuals, killing ~ 59,000 people annually, and causes at least USD 8.6 billion in economic losses annually [62]. In summary, rabies is a well understood, data-rich disease with a high degree of host plasticity and a demonstrated history of viral conservatism across host lineages, resulting in disease persistence. These elements make the rabies system a unique opportunity for advancing the global understanding of spillover transmission across regions, host taxa, and periods.

### Spillover transmission of rabies virus

**Fig. 5:**
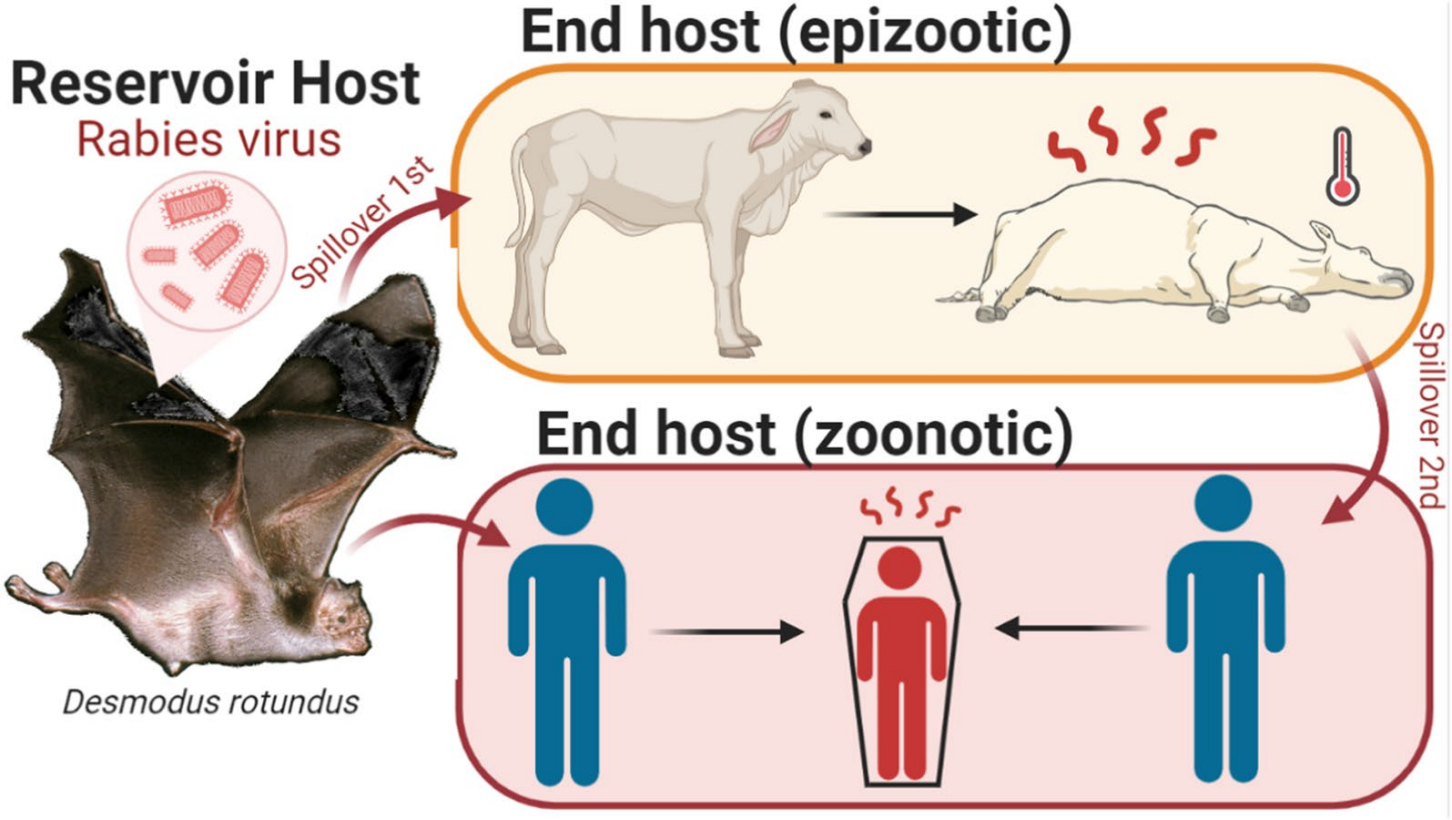
*Desmodus rotundus* rabies virus spillover and onward transmission. Rabies virus is transmitted accidentally from one infected vampire to its prey Rabies virus is transmitted from an infected vampire bat (i.e., reservoir host) to other species, which may or may not maintain transmission (i.e., end host vs. new host; Fig. 3). Primary spillover transmission events are common in Latin America. Secondary spillover transmission also occurs and result in zoonotic spillover (i.e., human infections)

While canine-rabies has been almost eradicated in the Americas, bat-borne rabies is now an emerging public health and agricultural problem [56, 63]. In Latin America, most rabies cases in humans and domestic animals originate from bites inflicted by the common vampire bat (*Desmodus rotundus*) (Fig. 5), one of the only three mammal species 100% dependent on blood to survive [56, 63]. The species has a broad geographic range, and feeds on the blood of a variety of prey species, including wildlife, livestock, pets, and humans [64]. Some of the southernmost *D. rotundus* populations in South America even feed on marine mammals (e.g., sea lions) and birds (e.g., penguins). During feeding, *D. rotundus* can transmit rabies virus to their prey (Fig. 5), which may result in primary spillover transmission or even secondary spillover to other species (e.g., rabies from *D. rotundus* to cats and then from cats to humans). Vampire bat-borne rabies virus (VB-RABV) outbreaks in humans suggest that mortality mainly occurs in tropical and subtropical regions. For example, Brazil reports about two cases of human rabies annually caused by bat-borne rabies viruses that have been laboratory-confirmed as antigenic variant 3, a RABV variant associated with *D. rodundus*. However, underreported cases that are not laboratory confirmed are probably much more common. Documenting how VB-RABV can spill from *D. rotundus* over to other species can inform and guide strategies to prevent VB-RABV spillover transmission to humans and domestic animals, and help prevent the potential spread of *D. rotundus* rabies from Latin America into the United States.

**Fig. 6:**
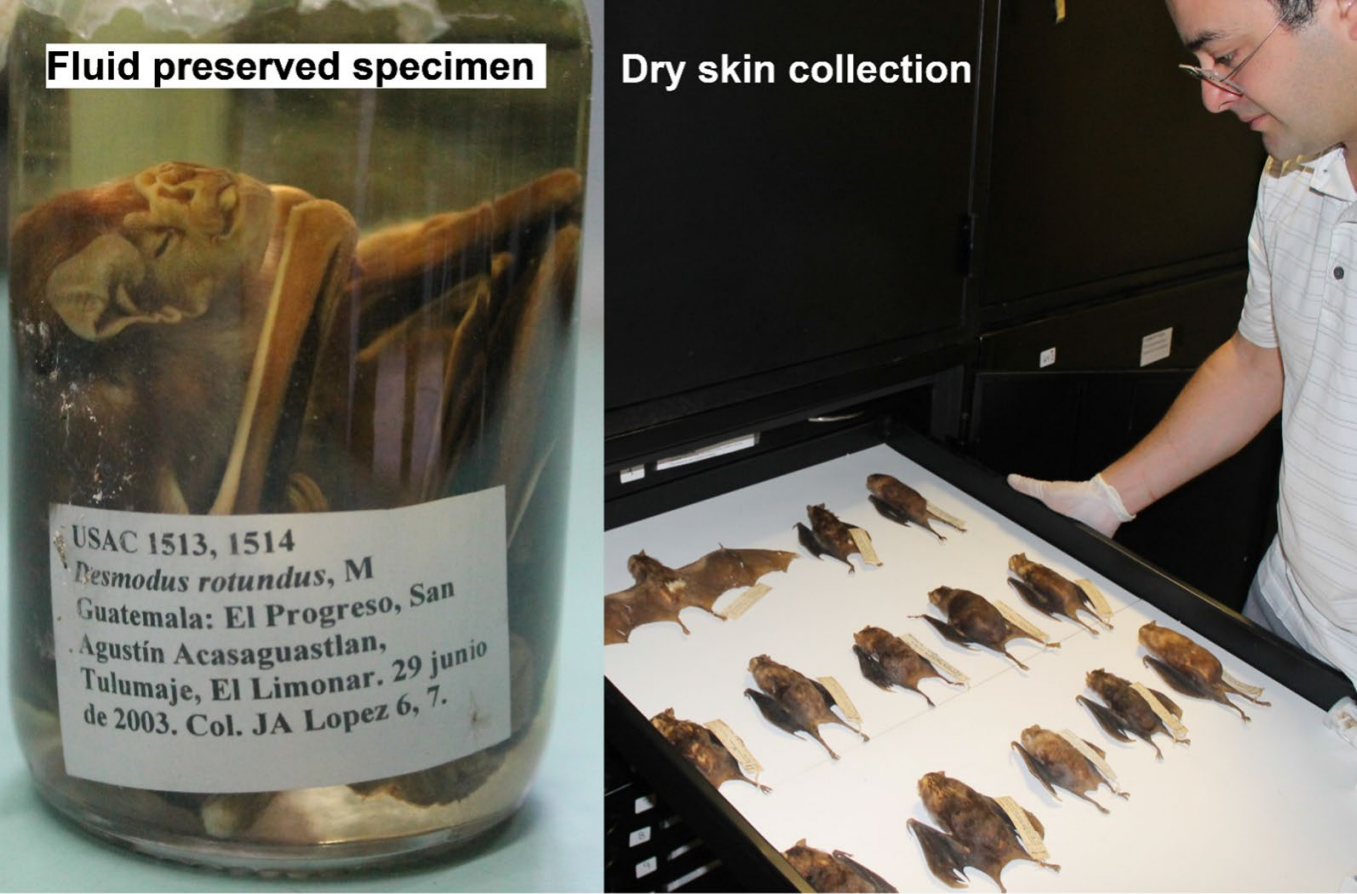
Large vampire bat records. Unpublished records of *Desmodus rotundus* and specimens are available across Latin America. For example, specimens are stored in biological collections in Latin America and available for future research to study spillover transmission and disease emergence

In summary, VB-RABV has been documented for over a century [65], and as a system displays many interesting elements of spillover transmission. VB-RABV from wildlife species is regularly transmitted to domestic animals and humans [64, 66], has a conservative virus for which dramatic viral genomic change has not been observed during some spillover events [61], and possesses an original reservoir host species (bats from the New World) which is often times evolutionary distant from its receptor hosts (e.g., herbivores from the Old World) [60]. Furthermore, *D. rotundus* is one of the most well studied mammals in the Americas [57, 58, 64, 67] (Fig. 6; considerable availability of specimens of the species), and is distributed across tropical regions where well-studied species are scarce. Comprehensive documentation and available data on rabies virus and the vampire bat geographic distribution across the Neotropics is reasonably well collected in a continuous and standardized form. That is, bat hosts and virus detection and identification are attained with high certainty by a continent-wide comprehensive surveillance system. In Latin America alone, at least 23,536 outbreaks of VB-RABV spillover to cattle were reported between 1970 and 2021 [68]. For example, VB-RABV lineage antigenic variant 3, specific to *D. rotundus*, shows failure to establish onward transmission after spillover transmission to livestock. These elements make vampire bat borne rabies an extraordinarily unique wildlife-disease system for advancing the understanding of spillover transmission across large study areas.

## Conclusions

Virus spillover transmission, as the prelude for disease emergence, is a poorly understood highly complex phenomenon [10]. Although spillover transmission is expected to increase in incidence and geographic range in the future years as a result of global change [6], the role of environmental factors at different scales of complexity has been rarely studied quantitatively [69]. As a result of these uncertainties, there is a poor mechanistic understanding of spillover transmission inherent processes (subtle stages) that limits humanity’s ability to predict virus spillover transmission across different regions. A persistently unsolved question in disease ecology, therefore, is the extent to which spillover transmission can be quantified and predicted at the local, regional, and continental levels (i.e., across spatial scales). Research is still needed on the ecological and biogeographic drivers leading to cross-species viral transmission to determine the mechanisms that facilitate host-virus dynamics across regions and environmental gradients [70–72]. Understanding where spillover transmission events are more likely to occur is the crucial first step where focused surveillance should take place, to anticipate effective early prevention and control programs *before* virus spillover transmission ends in disease emergence. The *status quo* in spillover transmission research reveals predictive limitations that fail to determine when a spillover transmission event could result in outbreaks, epidemics, and pandemics such as the COVID-19 pandemic caused by SARS-CoV-2.

Vampire-bat-borne rabies (VB-RABV) is an ideal biological system to study spillover transmission. For instance, RABV is well understood and there is an effective vaccine to reduce biosecurity risks for researchers. Additionally, VB-RABV spillover from *D. rotundus* to livestock is frequent and widespread in Latin America. Finally, VB-RABV primary spillover transmission to cattle results in end hosts, so that there are immense opportunities to better understand actual spillover transmission events that do not necessarily result in the establishment of new transmission cycles that may end up in long-lasting epidemics.

Future research in this field should focus on five key questions. (1) What landscape conditions are needed for a spillover transmission event to occur in the first place? (2) How can biodiversity composition modulate the likelihood of spillover transmission? and (3) What type of mutations in viral genomes facilitate spillover transmission and subsequent disease spread across different species? (4) Are there any climatic drivers favoring or limiting spillover transmission? (5) How do landscape changes, such as changes due to emergent commodity or natural resources exploitation (*e.g.,* lithium) or massive destruction after armed conflicts impact spillover transmission of wildlife viruses? These research lines are of critical public interest considering that the circulation of zoonotic viruses in wildlife is, in general, a threat to human health and social/economic development [73]. By understanding the specific drivers of zoonotic virus spillover transmission from wildlife, and by forecasting areas suitable for successful spillover transmission, health professionals could implement early detection, control, and elimination strategies for effective outbreak containment that will reduce economic impacts.

## Data Availability

Not applicable.

## Abbreviations

RABV: Rabies virus
VB-RABV: Vampire Bat Rabies or Vampire Bat-borne Rabies
US: United States
